# Distribution Characteristics and Source Analysis of Nitrogen and Phosphorus in Different Rivers in Two Water Period: A Case Study of Pi River and Shiting River in the Upper Reaches of Tuo River in China

**DOI:** 10.3390/ijerph191912433

**Published:** 2022-09-29

**Authors:** Tongfei Li, Pingyan Zhou, Yunchang Ding, Qiding Tang, Shanshan Zhou, Ying Liu

**Affiliations:** 1 College of Life and Environmental Sciences, Minzu University of China, Beijing 100081, China; 2 Beijing Engineering Research Center of Food Environment and Public Health, Minzu University of China, Beijing 100081, China

**Keywords:** nitrogen and phosphorus fractions, distribution characteristics, source analysis, Pi river, Shiting river, land use type

## Abstract

In this paper, the distribution characteristics of total nitrogen (TN), total phosphorus (TP) and fractions of nitrogen and phosphorus in water and surface sediments of the Pi and Shiting rivers in the dry and wet seasons were studied by molybdenum blue/ascorbic acid spectrophotometry and Standard Measurements and Testing (SMT). Correlation analysis, cluster analysis and principal component analysis were used to identified nitrogen and phosphorus pollution sources. The results showed that: (1) nitrogen and phosphorus in water and surface sediments in the study area were at different levels. (2) In the Pi river, the decomposition of animal and plant residues, the leachate from the accumulation of aquaculture wastewater and urban domestic sewage were the main sources of nitrogen and phosphorus pollution, while in the Shiting river, the unreasonable application of pesticides and fertilizers, the degradation of animal and plant residues, agricultural wastewater from agricultural drainage channels, industrial production wastewater and the weathering of agricultural wastes had a great impact on the nitrogen and phosphorus pollution. The results in this study provide reliable experimental data and a reference to local relevant departments for the implementation of effective control measures for the reduction of the nitrogen and phosphorus pollution load in the river basin.

## 1. Introduction

With the rapid growth of the economy, the discharge of industrial and domestic sewage in river systems is increasing, together to nitrogen and phosphorus pollution, leading to eutrophication and the deterioration of water quality [1]. Different fractions of nitrogen and phosphorus have different effects and feedback on the environment. Different pollution sources of nitrogen and phosphorus will lead to different proportions of their fractions in water and have great effects on aquatic organisms [2]. Furthermore, influenced by natural and social factors such as land types, watershed characteristics, pollutant sources and hydrological processes, the spatial distribution and pollution sources of nitrogen and phosphorus in rivers are obviously different. 

The Tuo river is a tributary of the Yangtze river in China and one of the most important river systems in the hinterland of the Sichuan basin. At the same time, the upper reaches of Tuo river are an important heavy industrial and mineral area in Sichuan Province. The unreasonable exploitation of phosphate rock and factory drainage have resulted in high contents of total phosphorus in the water and surface sediments. Although the concentrations of total phosphorus and nitrogen in the Tuo river have been decreasing in recent years [3], the river basin has always been difficult to clean up, and the pollution load is still in a serious overloading state [4]. The Shiting and Pi rivers are two important tributaries upstream of the Tuo river [5]. The land use type in the catchment area of the Pi river is mainly urban land, although some areas are agricultural land. Garbage, livestock and poultry excrement, pesticides and fertilizers are washed into the river with rainwater. In the upper reaches of Shiting river basin, the land use type is mainly forest, while the middle and lower reaches are mainly agricultural land. Industrial areas are distributed near the river. The output of phosphate ore in the lower reaches accounts for more than 95% of the output in Sichuan Province [5]. At the same time, the watershed is an important agricultural planting base in Chengdu Plain. It uses thousands of tons of chemical fertilizers and pesticides every year [4]. As the vast majority of local agricultural irrigation water comes from the river, after irrigation, the water flows back into the river, leading to increasingly prominent environmental problems. 

In recent years, many studies have sought to identify the composition of nitrogen and phosphorus pools and their roles in pollution. The authors of [6] studied the composition and structural characteristics of dissolved phosphorus, particulate phosphorus and organic P of algae in Tai Lake and Chao Lake, i.e., two eutrophic lakes in China. The factors of influencing the migration and transformation in lake ecosystems were also investigated. The results can provide an important theoretical basis for the mutual conversion process of organic phosphorus components between various media in the lake water environment [6]. The authors of [7] evaluated the relationship between suspended sediment and total phosphorus in river runoff of different land use modes in Japan. The results indicated that merely using total phosphorus as a substitute for bioavailable phosphorus was questionable, which overestimated the eutrophication potential of some phosphorus sources [7]. The authors of [8] analyzed the concentrations of different fractions of nitrogen and put forward reasonable suggestions for improving the water quality in the Wei river basin [8]. By studying the contributors to nitrogen loss in sediment cores in coastal bays of Daya Bay, the authors of [9] found that nitrification and denitrification or coupling of anammox has a large potential to contribute to the removal of buried or transported N that cannot be completely removed from surface sediments [9]. Over the past few years, the influence of the basin pattern structure, social economy and agricultural activities on river water quality have been receiving growing levels of attention [10]. However, due to the influence of external factors such as the current land use, soil composition, point source pollution, human activities and the social and economic development level in the basin, the correlation between land use and water quality is cancelled out or masked [11]. In recent years, few studies have been published on the relationship between land use and the evolution of river water pollution. At present, scholars are mostly focusing on the distribution characteristics and change patterns of different fractions of nitrogen and phosphorus in water or surface sediments in a single lake or river, or separately studying the distribution patterns of different fractions of nitrogen or phosphorus and predicting the development and change of nitrogen and phosphorus in the future by combining different models. However, there is a lack of comparative analyses and evaluations of sources for different fractions of nitrogen and phosphorus in water and surface sediments of different rivers in different water periods, and targeted solutions are rarely put forward. Therefore, we believe that the distribution characteristics and pollution levels of total nitrogen and phosphorus and their fractions in the water and surface sediments in different periods and under different land use types may be different, and that the pollution sources of different rivers may vary greatly. Exploring this scientific problem has important theoretical and practical significance for analyzing the pollution sources of rivers and devising targeted pollution control strategies.

This study took the Pi and Shiting rivers in the upper reaches of Tuo river as the research objects, (1) to explore the distribution rules of total nitrogen, total phosphorus and fractions of nitrogen and phosphorus in the water and surface sediments, and to compare and study the distribution patterns of nitrogen and phosphorus pollution in different water periods with different land use types; and (2) to analyze the sources of nitrogen and phosphorus pollution using correlation analysis, principal component analysis and cluster analysis. The present study may provide a reliable basis for the prevention and control of nitrogen and phosphorus pollution in the watershed. 

## 2. Materials and Methods

### 2.1. Description of the Study Area

**Figure 1:**
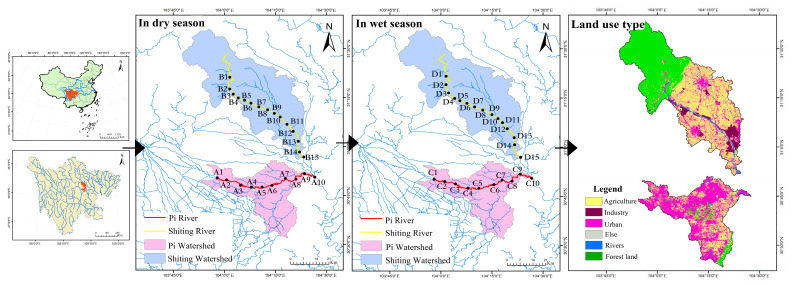
Distribution of sampling sites and land types in different water periods (The data set of Chinese Map was provided by the Data Center for Resources and Environmental Sciences, Chinese Academy of Sciences (http://www.resdc.cn, accessed on 10 March 2021)).

The Shiting and Pi rivers, two important tributaries of the upper reaches of the Tuo river in Sichuan Province, China, were selected for this study. Water and surface sediments samples were collected from the two rivers in February 2021 (dry season) and July 2021 (wet season). The distribution of sampling sites and land types in different water periods is shown in Figure 1. The Pi river originates in Shidiyan, Unity Town, Pidu District, Chengdu City, Sichuan Province; upstream is the Baitiao river of the Dujiangyan river system [12]. The topography of the catchment area is high in the northwest and low in the southeast, and the ground slopes steeply. The length of the river is about 65 km [13], its average width is 50–100 m, its depth is 3.5 m, the elevation is 350–450 m, the annual average flow is about 26 m^3^·s^−1^ and the average precipitation is 1200 mm. The main land use type is urban, with some areas being agricultural. The irrigated area of the watershed is 238 km^2^ and aquaculture and planting are well developed. The population of the urban area around the basin reaches 4.48 million. Household garbage, livestock manure, pesticides and fertilizers often wash into the river following rain. The Shiting river is one of the headwaters of the Tuo river, a tributary of the Yangtze river [5]. Forest is the main land use type in the upper reaches of the catchment, while agricultural land is the main use type in the middle and lower parts of the river, which flows through Deyang City, an important phosphorus chemical industry base in China. Most of the phosphorus mining enterprises are located in the Mianyuan and Shiting river basins [14]. Over the decades, this has led to increasing environmental problems. Because there are a large number of phosphorus chemical enterprises (more than 80 large-scale operations) along the Shiting river, the phosphogypsum resulting from the production process is mostly piled up along the river bank. Since many old phosphogypsum dumps have not taken measures such as anti-seepage and leachate collection, under the influence of rainfall, industrial waste liquid, waste gas and waste residue easily to flow into the river [15]. In addition, the watershed is an important agricultural planting base in the Chengdu plain. Rice, wheat and vegetables are the main crops on both sides of the river, covering an area of more than 5100 acres. The annual use of chemical fertilizers and pesticides is about 1000 tons [16]. Chemical fertilizers contain a lot of nitrogen, phosphorus, organic matter and heavy metals, and only 35% is adsorbed by plants as intended. Most of the local agricultural irrigation water is taken from the river, and the backwater after irrigation joins the river again, so the water quality of the Shiting river is seriously threatened.

### 2.2. Determination and Quality Control of Nitrogen and Phosphorus Fractions

**Figure 2:**
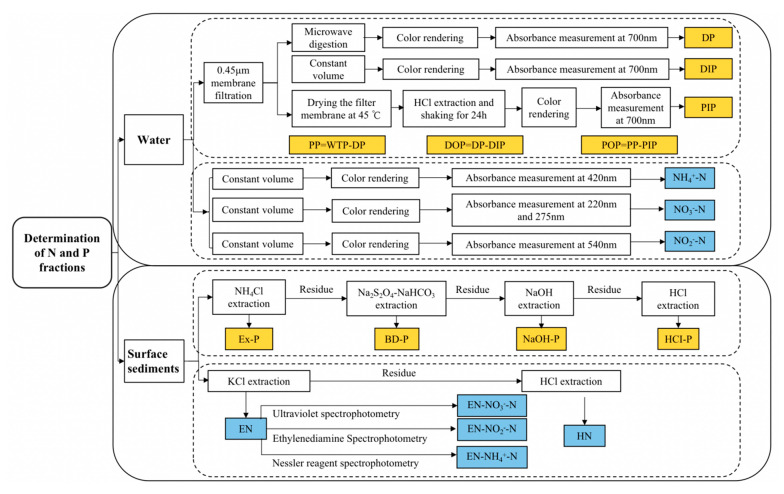
The determination steps of N and P fractions in water and surface sediments.

In our water samples, total phosphorus (WTP), dissolved phosphorus (DP) and particulate phosphorus (PP) were determined according to the National Standard (GB 11893-89) and molybdenum blue/ascorbic acid spectrophotometry [17,18]. Total nitrogen (WTN), ammonia nitrogen (NH_4_^+^-N), nitrate nitrogen (NO_3_^−^-N) and nitrite nitrogen (NO_2_^−^-N) were determined according to National Standards (GB 11894-89), (HJ535-2009), (HJ/T 346-2007) and (GB 7493-87) [19,20,21,22]. The measurement steps are shown in Figure 2. 

In surface sediments, total phosphorus (STP) and inorganic phosphorus (IP) were measured according to Standard Measurements and Testing (SMT) [23]. Weakly exchangeable phosphorus (Ex-P), reducible phosphorus (BD-P), metal oxide-bound phosphorus (NaOH-P) and calcium-bound phosphorus (HCl-P) were determined by continuous extraction, following the method proposed by Hupfer [24] and Psenner et al. [25]. The total nitrogen was determined by alkaline potassium persulphate digestion [26]. Each fraction of nitrogen was extracted using the continuous extraction method to obtain exchangeable nitrogen (EN) and acidolytic nitrogen (HN) [27], and the contents of NH_4_^+^-N, NO_3_^−^-N and NO_2_^−^-N in EN and HN were determined using the national standard methods described previously.

The phosphorus standard solution, nitrate nitrogen standard solution and ammonia nitrogen standard solution used in the experiment were purchased from the National Institute of Nonferrous Metals. The standard material for stream sediment analysis (GBW07307a) was purchased from the Institute of Geophysical and Geochemical Exploration, Chinese Academy of Geological Sciences. According to our standard addition recovery experiment, the recovery rates of TP, TN, NH_4_^+^-N, NO_3_^−^-N and NO_2_^−^-N in water were 96.7–99.8%, 94.8–95.3%, 90.8–98.7%, 92.3–94.1% and 89.9–94.6%, respectively. The recovery rates of TP and TN in surface sediments were 94.6–95.4% and 97.1–102%, respectively, and the coefficient of variation was less than 10%.

## 3. Results and Discussion

### 3.1. Distribution Characteristics of Total Phosphorus Concentration and Pollution Levels in Water

**Figure 3:**
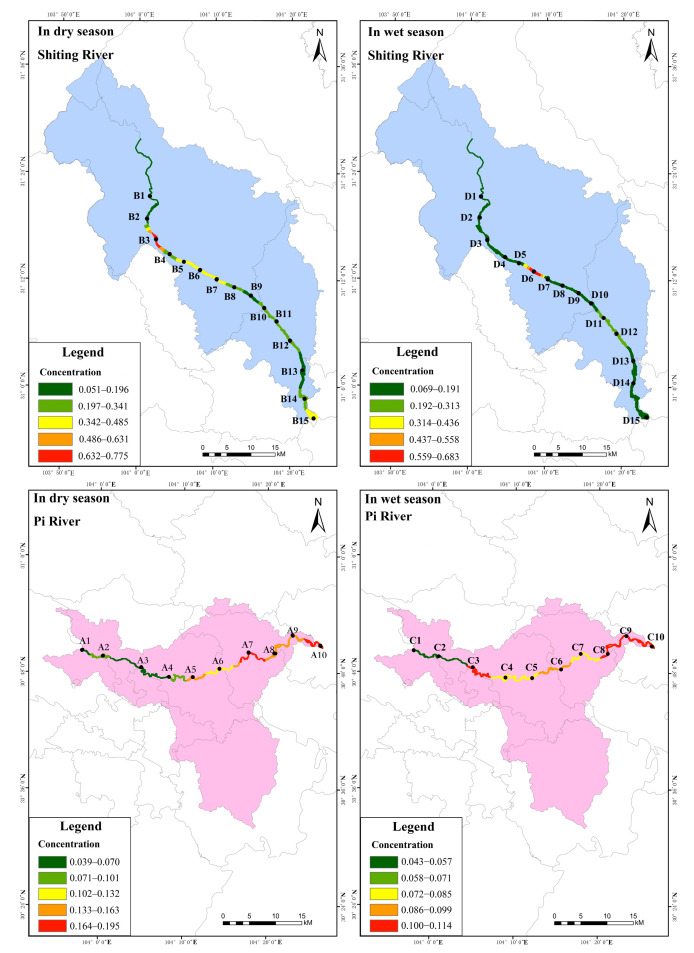
Spatial distribution of TP in water of the study area.

The distribution characteristics of total phosphorus (WTP) concentration in the water of the study area are shown in Figure 3 (Table S4). As shown, in the dry season, the WTP concentration in the Pi river showed low levels in the upstream area, gradually increasing downstream, with concentrations in the range of 0.039–0.195 mg·L^−1^ and a mean value of 0.120 mg·L^−1^. Among them, the concentration of the WTP at sampling point A10 was significantly higher. This site was located in the lower reaches of the river, where municipal solid waste and livestock manure are discharged. Additionally, this point was located where the Pi river converges with the Fu river on its way to the Tuo river [28], and as such, the phosphorus pollution was partly from Pi and partly from Fu. Because the Fu river is also an inland river, the planting and aquaculture industries along it are quite developed, and as such, the phosphorus concentration in the river was higher. In accordance with China’s Environmental Quality Standards for Surface Water [29] (Table S1) and the Specification for Eutrophication Investigation [30] (Table S2), the water quality at points A1, A3 and A4 in the Pi belonged to Class IV, while point A5–A10 belonged to Class V; points A1 and A2 were eutrophic, while points A4–A10 were heavy eutrophic. 

It can also be seen from Figure 3 that in the dry season, the WTP concentration in the Shiting river increased gradually from the upstream to the midstream and decreased in the downstream. Moreover, the WTP concentration at different sampling points varied greatly, ranging from 0.051 to 0.776 mg·L^−1^, with an average of 0.292 mg·L^−1^, Notably, there are a large number of phosphorus chemical enterprises in the vicinities of sampling points B5–B7. Due to factors such as leaching and the heavy discharge of industrial wastewater [31], phosphorus concentrations in the water at these points were high. However, downstream sampling points B8–B13 were far from the dense industrial areas, and the phosphorus concentration decreased through the dilution of the river. In contrast, sampling points B14 and B15 were located near the intersection of the Shiting river, the Yazi river and the Mianyuan river into the Tuo river. In the middle and lower reaches of Mianyuan and Shiting rivers, there are many phosphate mining enterprises. The Yazi river basin is a drainage and irrigation site [32] which passes through urban areas, resulting in a wide range of phosphorus pollution sources and high phosphorus concentrations at sampling points B14 and B15. Our study indicated that except for point B1, which showed Class IV water quality and eutrophic type, all other points showed Class V water quality and heavy eutrophic type.

In the wet season, the WTP concentration in the Pi river increased slowly from upstream to downstream, ranging from 0.043–0.114 mg·L^−1^, with an average value of 0.085 mg·L^−1^. According to China’s Environmental Quality Standards for Surface Water [29] and the Specification for Eutrophication Investigation [30], sampling points C3, C8, C9 and C10 showed Class V water quality and heavy eutrophic type. In the Shiting river, except for point D6, all other sampling points showed a gradually increasing distribution pattern from upstream to downstream, and the WTP concentration at each sampling point was in the range of 0.069–0.683 mg·L^−1^, with an average of 0.177 mg·L^−1^. The water quality of D7–D15 in the Shiting river was Class V; D4 and D7–D15 were heavy eutrophic type, and all other sampling points were Class IV. The reasons for the higher WTP in the downstream of the Shiting river compared to the upstream may be that the farmland in the catchment area is widely and densely distributed [33], and the fertilizers and pesticides used there are washed into the river by precipitation [34]. Therefore, it is necessary to pay attention to seriously polluted areas according to the distribution pattern of WTP concentrations. The WTP concentration in the dry season was higher than that in the wet season due to the low runoff; as such, it is important to pay more attention to the pollution in the two rivers in the dry season. In addition, in the dry season, points A9 and A10 in the lower reaches of the Pi river and B5–B7 in the middle and lower reaches of the Shiting river should be monitored regularly. Meanwhile, in the wet season, the lower reaches of the Pi river and D6, D13–D15 in the Shiting river should be used as key areas for pollution monitoring.

### 3.2. Distribution Characteristics of Total Nitrogen Concentration and Pollution Levels in Water 

**Figure 4:**
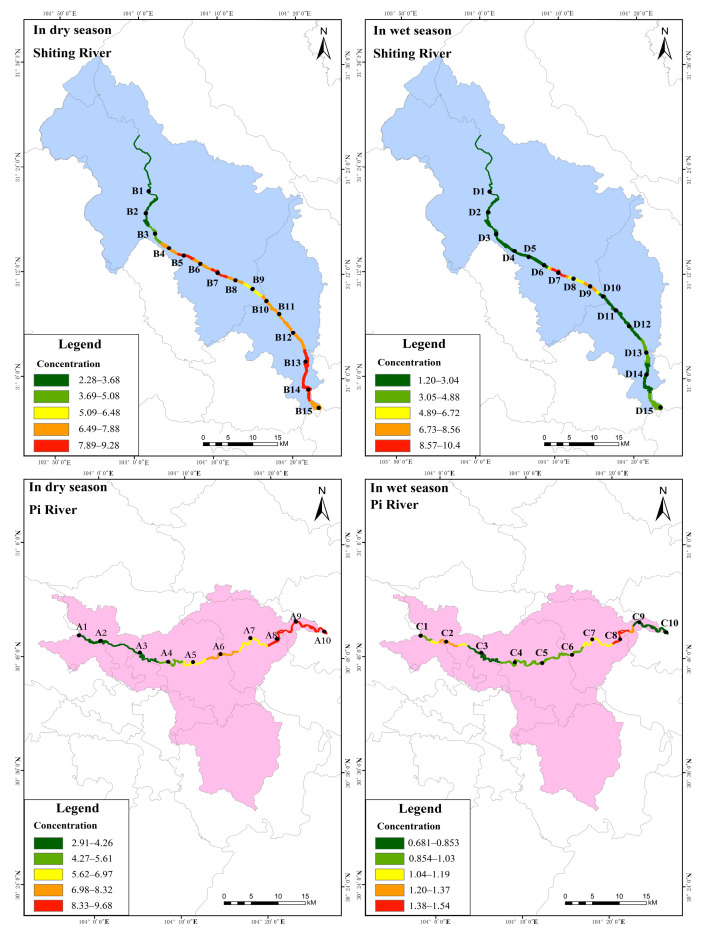
Spatial distribution of TN in water of the study area.

The distribution characteristics of total nitrogen (WTN) concentration in the water of the study area are shown in Figure 4 (Table S4). As shown, in the dry season, the WTN concentration in the Pi river showed a distribution pattern of low upstream and gradually increasing downstream. The WTN concentration was in the range of 2.91–9.68 mg·L^−1^, with an average of 6.13 mg·L^−1^. The high concentration of WTN in the downstream was likely caused by the accumulation of urban domestic pollution, such as livestock manure and farming wastewater, which were the main sources of nitrogenous pollutants [35]. In addition, aquaculture and fishing activities resulted in the presence of more bait containing nitrogen being put into the river. It was found that the concentration of WTN in the Shiting river showed a gradually increasing distribution pattern from the upstream to middle and then downstream, with concentrations in the range of 2.28–10.9 mg·L^−1^, and an average of 6.95 mg-L^−1^. According to the China’s Environmental Quality Standards for Surface Water [29] and the Specification for Eutrophication Investigation [30], all sampling points in the Pi and Shiting rivers were heavy eutrophic type and had Class V water quality in the dry season. 

Figure 4 also shows that in the wet season, the WTN concentration in the Pi river varied little from the upstream to the downstream, ranging from 0.681–1.54 mg·L^−1^, with an average value of 0.993 mg·L^−1^, while the WTN concentration in the Shiting river first showed an increasing trend, then a slight decrease, and then an increase, with the WTN concentration in the range of 1.20–10.4 mg·L^−1^ and an average of 3.27 mg·L^−1^. According to China’s Environmental Quality Standards for Surface Water [29] and the Specification for Eutrophication Investigation [30], the C8 sampling site in the Pi river in the wet season was of Class V water quality, C2 and C7 were of Class IV, C2 and C8 were of heavy eutrophic type, and all other points were below Class IV. However, D4, D6–D9, D11, and D13–D15 in the Shiting river were all Class V, while the rests of the sites were Class IV. Except for point D2, which was eutrophic, all other sites were of the heavy eutrophic type. The WTN pollution in the wet season was lower than that in the dry season. It was supposed that the higher runoff in the wet season led to faster flow, deeper waters, river erosion, and lower concentrations of pollutants after dilution [36]. The WTN in the middle reaches of the Shiting river was high; this was attributed to the presence of the Xinshi Industrial Park and the Equipment Manufacturing Park in this area. The main discharge outlet of the Xinshi Industrial Park in Mianzhu City is located nearby, causing a large amount of wastewater to flow into the river. It was recently reported that illegal discharge and leakages were occurring nearby [37], causing serious nitrogen and phosphorus pollution at this point. Therefore, it is necessary to focus on the key pollution areas according to the distribution pattern of WTN concentration. For example, in the dry season, sampling points A8–A10 in the lower reaches of the Pi river and B4–B8 and B13–B15 in the middle and lower reaches of the Shiting river should be regarded as the key monitoring areas. In the wet season, the lower reaches of the Pi river and D7–D9 on the Shiting river should be treated as the key areas for monitoring WTN pollution.

### 3.3. Distribution Characteristics of Nitrogen and Phosphorus Fraction Concentrations and Pollution Levels in Water 

**Figure 5:**
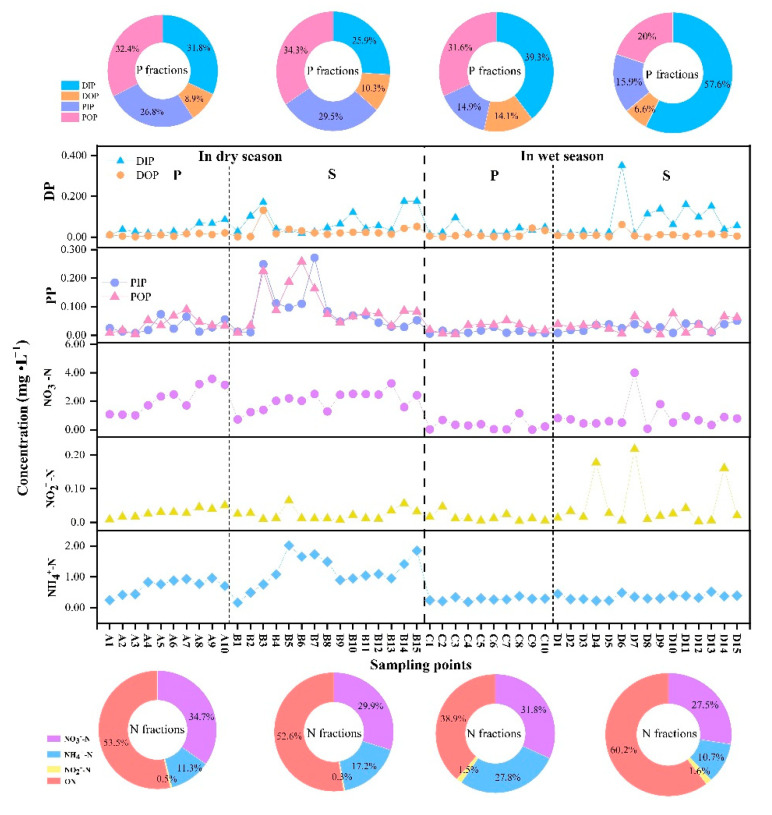
N and P fraction concentration distribution in water in the study area. (P: Pi river, S: Shiting river).

The distribution characteristics of the nitrogen and phosphorus fraction concentrations in the water of the study area are shown in Figure 5 (Table S4). Phosphorus in the water can be divided into four fractions: dissolved inorganic (DIP), dissolved organic (DOP), particulate inorganic (PIP) and particulate organic (POP) [38]. As shown, in the dry season, the concentrations of DIP and DOP in the the Pi river were relatively low and close to each other, while the concentrations of PIP and POP in the middle and lower reaches were quite different. The average values of the four fractions of phosphorus were in the following order: POP (0.039 mg·L^−1^) > DIP (0.038 mg·L^−1^) > PIP (0.032 mg·L^−1^) > DOP (0.011 mg·L^−1^), where POP, DIP, and PIP were significantly higher than DOP. Meanwhile, the average values of these fractions in the Shiting river were POP (0.10 mg·L^−1^) > PIP (0.086 mg·L^−1^) > DIP (0.075 mg·L^−1^) > DOP (0.030 mg-L^−1^). In the wet season, the average values of four phosphorus fractions in Pi river were DIP (0.033 mg·L^−1^) > POP (0.026 mg·L^−1^) > PIP (0.012 mg·L^−1^) > DOP (0.011 mg·L^−1^), and in the Shiting river, the average values were DIP (0.088 mg·L^−1^) > POP (0.035 mg·L) > PIP (0.028 mg·L^−1^) > DOP (0.011 mg·L^−1^). 

The effect of dissolved phosphorus (DP) on water eutrophication was significant [39]. A comparison showed that in the wet season, the DP concentrations in the two rivers were higher than in the dry season, and eutrophication was more likely to occur. In addition, it was also found that the DP concentration of the Pi river in the wet season was higher than that in the dry season, presumably due to the higher runoff (up to 80 m^3^·s^−1^) in the wet season. Additionally, river scouring led to the suspension of a large number of particles. Compared with the low flow rate, the proportion of coarse particles transported by the high flow rate was higher, and the suspended particles contained less particulate phosphorus and more dissolved phosphorus [7]. However, the DP in the Shiting river was higher than that in Pi river in the dry and wet seasons. As the agricultural cultivation area of the Shiting river comprises over 5100 acres, the dissolved phosphorus in the soil was accumulated due to the application of fertilizer and manure [40] which was washed into the river by precipitation. This indicated that the control of phosphorus pollution in the Shiting river should be strengthened. 

The nitrogen fraction in water includes nitrate-nitrogen (NO_3_^−^-N), nitrite-nitrogen (NO_2_^−^-N), ammonia-nitrogen (NH_4_^+^-N), and organic-nitrogen (ON). Among them, NO_3_^−^-N, NO_2_^−^-N, and NH_4_^+^-N are the most active fractions, and are easily absorbed and then utilized by aquatic organisms [41,42]. Figure 5 shows that in the dry season, the average values of the four nitrogen fractions in there Pi river were in the following order: ON (3.28 mg·L^−1^) > NO_3_^−^-N (2.13 mg·L^−1^) > NH_4_^+^-N (0.693 mg·L^−1^) > NO_2_^−^-N (0.029 mg·L^−1^). Meanwhile, those in the Shiting river were in the following order: ON (3.59 mg·L^−1^) > NO_3_^−^-N (2.04 mg·L^−1^) > NH-N (1.17 mg·L^−1^) > NO_2_^−^-N (0.023 mg·L^−1^). In the wet season, mean concentrations of the four nitrogen fractions in the Pi river were in the following order: ON (0.386 mg·L^−1^) > NO_3_^−^-N (0.317 mg·L^−1^) > NH_4_^+^-N (0.277 mg·L^−1^) > NO_2_^−^-N (0.015 mg·L^−1^). Meanwhile, those in Shiting river were in the following order: ON (1.97 mg·L^−1^) > NO_3_^−^-N (0.899 mg·L^−1^) > NH_4_^+^-N (0.350 mg·L^−1^) > NO_2_^−^-N (0.052 mg·L^−1^). 

In comparison, the concentrations of NO_3_^−^-N and NH_4_^+^-N in the two rivers showed a similar trend to that of WTN in the dry season, while the concentrations of NO_2_^−^-N were lower. The concentrations of NO_3_^−^-N, NO_2_^−^-N, and NH_4_^+^-N in the Pi river increased gradually from upstream to downstream, while the concentrations in the Shiting river increased rapidly in the upstream and slightly decreased in the middle and downstream. In the wet season, the concentrations of NO_3_^−^-N and WTN in the Pi river had similar trends, while the concentrations of NH_4_^+^-N and NO_2_^−^-N were lower. However, the concentrations of NO_3_^−^-N and NO_2_^−^-N in the Shiting river were higher in the middle stream sampling points and lower in the upstream and downstream sampling points, and the concentration difference of NH_4_^+^-N at each sampling point was small. In addition, the proportion of ON in the Shiting river in dry season was lower than that in wet season. This was because the water temperature was about 10 °C, which is not conducive to the activities of submerged plants and phytoplankton, which were largely dormant at this stage, and as such, their utilization of nitrogen was low. However, the water temperature was about 20 °C in the wet season, and aquatic organisms and floating and submerged plants were active. Additionally, algae were abundant in the upper water, so the use efficiency of nitrogen was high [43], resulting in a low concentrations of the more biologically active fractions of nitrogen and a low concentration of WTN in the water. By comparing the variation patterns of nitrogen fractions in different periods in the two rivers, it was found that the concentrations of NO_3_^−^-N, NO_2_^−^-N, and NH_4_^+^-N, which were easily absorbed and utilized by algae, were higher in the Shiting river in dry season and in the Pi river in the wet season, while the concentrations of NO_3_^−^-N and NO_2_^−^-N in the middle reaches of the Shiting river in the wet season were higher. As such, this area should be used for monitoring. 

### 3.4. Distribution Characteristics of Total Phosphorus Content and Pollution Levels in Surface Sediments

**Figure 6:**
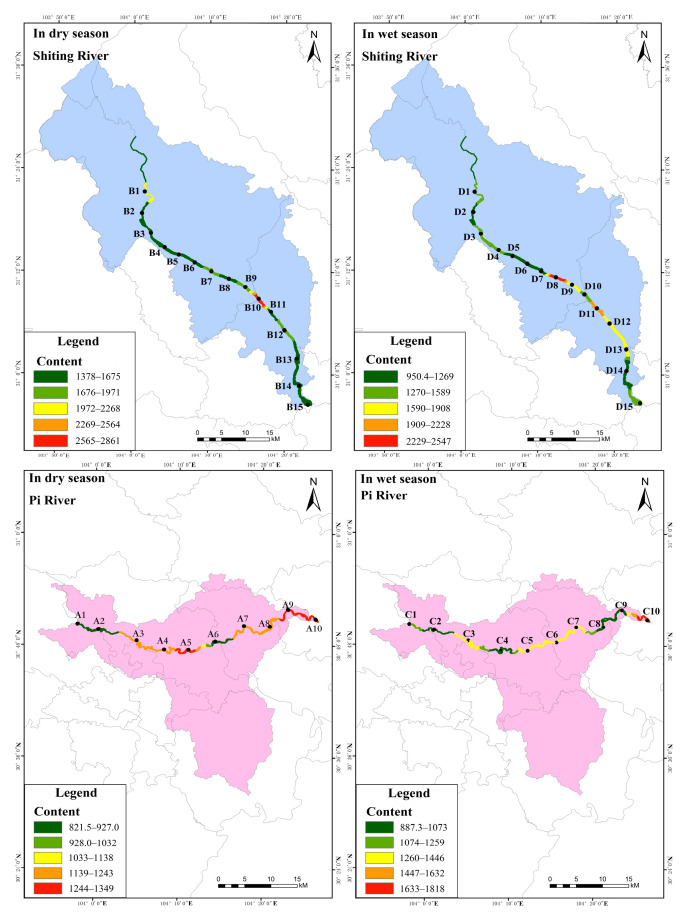
Spatial distribution of TP in surface sediments in the study area.

The distribution characteristics of total phosphorus content in the surface sediments (STP) of the study area are shown in Figure 6 (Table S5). As shown, in the dry season, the content of STP in the Pi river, except for that at point A6, gradually increased from the upstream to the downstream; the content range was 821.5–1349 mg·kg^−1^. As point A6 was located in a river reconstruction area, the hardening of the river ground allowed the surface sediments to be more readily washed by the water flow, and the pollutants were not inclined to accumulate, resulting in a low content. It is also clear from Figure 6 that the STP in the Shiting river showed a distribution of gradually increasing from the upstream to the midstream, and slightly decreasing in the downstream. The STP content varied less among these sampling sites, ranging from 1378–2861 mg·kg^−1^, with an average value of 1724 mg·kg^−1^. The upper reaches of the river were located in a transition region of land use type, i.e., from woodland to agricultural. This change in land use type led to a change of soil structure [44], which influenced the absorption and utilization degree of nitrogen and phosphorus of surface sediments. Therefore, the STP content at point B1 increased. According to the standards formulated by the US Environmental Protection Agency (EPA) [45] (Table S3), in the dry season, all surface sediment sampling points in the Pi and Shiting rivers were severely polluted. According to the standards of Environment Canada [46] (Table S3), in the dry season, sampling points B1 and B10 in the Shiting river could cause exhibit ecotoxic effects, while the other sampling points and all sampling points in the Pi river would likely display the lowest ecotoxic effects.

It can also be seen from Figure 6 that in the wet season, the STP content in the Pi river showed a distribution pattern with little change from the upstream to the middle reaches, decreasing first and then increasing in the downstream. The STP content range was 887.3–1818 mg·kg^−1^, with an average of 1139 mg·kg^−1^. It is speculated that in dry season, the Pi river was mainly a flood discharge channel, with large water flow and a wide river channel, and therefore, the surface sediments were scoured by the water flow, and the pollutants did not readily accumulate [47]. Point C10 was located in the Tuo river after the intersection of the Pi and Fuhe rivers. This sampling point was also located in Meilin Park, Jintang County. On the one hand, it was greatly influenced by human activities, which led to a significant increase in STP content, while on the other hand, it was located in a shallow bend in the river, and pollutants tended to accumulate in the surface sediments. However, the STP in the Shiting river showed a gradual increasing distribution from upstream to downstream, with content in the range of 950.4–2547 mg·kg^−1^, and an average of 1499 mg·kg^−1^. According to the standards formulated by the US EPA [45], all sampling points in the Pi and Shiting rivers were seriously polluted in the wet season. According to the standards of Environment Canada [46], sampling point D8 in the Shiting river could exhibit serious ecotoxicity in the wet season, while the other sampling points and all sampling points in the Pi river would likely be far less ecotoxic. Therefore, it is necessary to closely monitor seriously polluted areas according to the distribution of STP content. The STP content in the dry season was higher than that in the wet season because of the low level of runoff of the river. Therefore, it is necessary to focus on the phosphorus pollution in the surface sediments in the dry season.

### 3.5. Distribution Characteristics of Total Nitrogen Content and Pollution Levels in Surface Sediments

**Figure 7:**
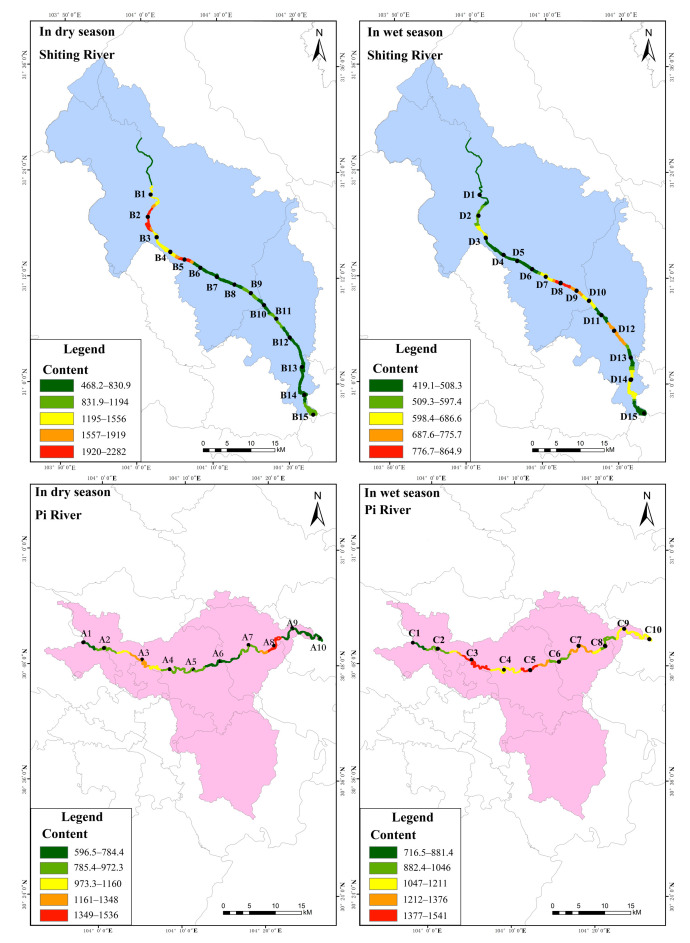
Spatial distribution of TN in surface sediments in the study area.

The distribution characteristics of total nitrogen (STN) content in the surface sediments of the study area are shown in Figure 7 (Table S5). As shown, in the dry season, the STN content in the Pi river showed a distribution pattern of increasing in the upper reaches and decreasing in the middle and lower reaches. The values ranged from 596.5 to 1536 mg·kg^−1^, and the average was 910.4 mg·kg^−1^. However, the STN in the Shiting river showed a gradually increasing distribution pattern from the upper to the middle reaches, with a content range of 468.2–2282 mg·kg^−1^ and an average value of 1073 mg·kg^−1^. The higher STN content in the Shiting river may be have been due to the wide and dense distribution of farmland in the catchment area, where a large amount of compound fertilizer is applied and the average utilization rate was found to be only about 35% [48]. As a source of agricultural irrigation, the Shiting river is used to irrigated crops. This water then flows back into the river through farmland drainage channels. After farmland is washed by water, nitrogen is lost, so STN content will be relatively high. According to the standards formulated by the US EPA [45], in the dry season, sampling points A3 and A8 in the Pi river and B2, and B5 in the Shiting river were severely polluted, while sampling points B1, B3, B4, B9, B11, and B15 were moderately polluted. All other points were mildly polluted. According to the standards formulated by Environment Canada [46], in the dry season, except for point B10, all sampling points in the Shiting river and Pi river would likely exhibit low levels of ecotoxicity.

It can also be seen in Figure 7 that in the wet season, except for points C3 and C4, the STN content in Pi river showed a distribution pattern of gradually increasing from the upstream to the middle reaches and then decreasing in the downstream. Its content was in the range of 716.5–1541 mg·kg^−1^, with an average value of 1116 mg·kg^−1^. Meanwhile, the STN content in the Shiting river showed a distribution pattern of slowly increasing from the upstream to the downstream. There was a small difference in STN content, with an average of 568.2 mg·kg^−1^ and a range of 419.1–864.9 mg·kg^−1^. By comparison, it was found that the STN content in the Pi river in the wet season was higher than that in the dry season. It was speculated that this was due to the large amount of arable land near the Pi river and the large number of bends in the river [47], causing nitrogen accumulation in the surface sediments. According to the standards formulated by the US EPA [45], except C1, C6, and C8 in the Pi river in the wet season, which exhibited mild pollution, all other points were moderately polluted. All sampling points in the Shiting river were slightly polluted. STN pollution in the Shiting river in the wet season was lower than that in the dry season. It was speculated that the high runoff in the wet season resulted in rapid water flow, significant river erosion nitrogen loss, and decreased STN content. According to the standards formulated by the Environment Canada [46], in the wet season, all sampling points in the Pi river, as well as D3, D7–D10, D12, and D14 in the Shiting river, exhibited the lowest ecotoxic characteristics. In conclusion, the nitrogen pollution in the surface sediments of the Pi river and the Shiting river was more serious, while that in the surface sediments of the Pi river was more serious than that of the Shiting river in the wet season. As such, the former should be used as a monitoring area.

### 3.6. Distribution Characteristics of Nitrogen and Phosphorus Fraction Contents and Pollution Levels in Surface Sediments

**Figure 8:**
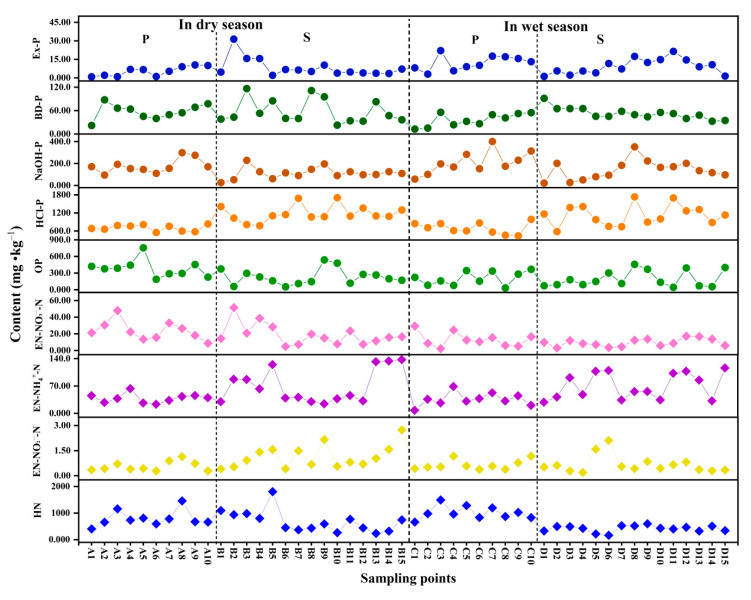
N and P fraction content distribution in surface sediments in the study area (P: Pi river, S: Shiting river).

The distribution characteristics of nitrogen and phosphorus fraction contents in surface sediments in the study area are shown in Figure 8 (Table S5). In this study, STP was classified into inorganic phosphorus (IP) and organic phosphorus (OP), according to the Standard Measurements and Testing (SMT) method [49], and was divided into weakly exchangeable phosphorus (Ex-P), reducible phosphorus (BD-P), metal oxide-bound phosphorus (NaOH-P), calcium-bound phosphorus (HCl-P), and residue phosphorus (Res-P), according to the modified Psenner method [24,25]. As shown, the trends for HCl-P and OP were similar to that for STP, and the trends of Ex-P, BD-P, and NaOH-P were comparable.

OP was found to be an important component of phosphorus in surface sediments, and its biochemical cycle played a key role in eutrophication [50]. In both the dry and wet seasons, the OP content in surface sediments in the Pi river was relatively high, accounting for 33.6% and 16.7% of STP, respectively, while that in the Shiting river was 13.4% and 12.9%, respectively. The STP content and its variation at each sampling point in the Shiting river were mainly influenced by IP, while OP was relatively stable. HCl-P, as an inert fraction of phosphorus in surface sediments, consisted of insoluble calcium carbonate minerals which are not readily absorbed by organisms [51]. It can also be seen from Figure 8 that in the dry and wet seasons, the average content of HCl-P in surface sediments in the Pi river were 61.6% and 56.4%, respectively, while that in Shiting river was 68.6% and 75.2%, respectively. This indicated that the content of HCl-P and the proportion of HCl-P in STP in the Shiting river were higher than that in the Pi river in the dry and wet seasons. The high content of HCl-P may have been due to the water environment in the Shiting river, which is influenced by an industrial park, making the water weakly alkaline for much of the time. Under such conditions, phosphorus can react with calcium to form precipitates which then settle into the sediment and are not readily released [52]. 

The sum of Ex-P, BD-P, and NaOH-P in surface sediments can be used to estimate the amount of bioavailable phosphorus (BAP) [53], i.e., that which can be directly absorbed and utilized by organisms, having a great effect on eutrophication of rivers. In the dry and wet seasons, the proportion of BAP in the surface sediments of the Pi river was higher, and the average proportion of BAP to STP was 20.9% and 20.6%, respectively, showing a higher phosphorus release risk and bioavailability and a greater effect on algal growth. The mean BAP to STP in surface sediments in the Shiting river was 11.0% and 13.9%, respectively. There are so many industrial areas in the middle and lower reaches of the Shiting river, and most of the phosphorus that is discharged into the river cannot be used by organisms. Therefore, different sources of phosphorus pollution in rivers with different land use types lead to different proportions of BAP in surface sediments and different consequences in terms of phosphorus pollution. 

The nitrogen fractions in surface sediments are mainly divided into exchangeable (EN), acidolytic (HN), and residual nitrogen (Res-N) [54]. Exchangeable nitrogen can be further divided into nitrate (EN-NO_3_^−^-N), nitrite (EN-NO_2_^−^-N) and ammonia (EN-NH_4_^+^-N). As shown in Figure 8, the changing trends of the three exchangeable nitrogen fractions of EN-NO_3_^−^-N, EN-NO_2_^−^-N and EN-NH_4_^+^-N were basically similar, while that of HN was similar to that of STN. HN can be hydrolyzed under strongly acidic conditions, mainly in the fraction of organic nitrogen, and can then be directly absorbed and utilized by plants and algae after mineralization [55]. Thus, HN indirectly effects the release of nitrogen from surface sediments. In both the dry and wet seasons, the distribution of HN was higher in the middle reaches and lower in the upstream and downstream of the Pi river. However, the content of HN in the Shiting river first increased and then decreased in the dry season. Meanwhile, in the wet season, the HN content showed little variation among all sampling sites, slowly increasing from the upstream to the downstream.

EN in surface sediments can be directly absorbed by primary producers for photosynthesis. It is also the most readily released fraction of nitrogen into the water [56]. In the dry and wet seasons, the average ratio of EN to STN in the Pi river was 7.23% and 4.60%, respectively, while that in Shiting river was 9.83% and 15.2%. Meanwhile, in the Shiting river, the proportion of EN in the surface sediments was relatively larger, and the nitrogen was more likely to be released into the water. Therefore, it is necessary to strengthen pollution monitoring of EN in the Shiting river in the wet season.

### 3.7. Ratio of Nitrogen to Phosphorus in the Water of the Pi and Shiting Rivers

WTN and WTP are often regarded as limiting indexes of algal growth. The ratio of N to P (N/P) is not only an important indicator of nutrient salt concentration in water; it also has a strong influence on the growth and extinction of algae in eutrophic water, which can indicate the nature of nutrient restriction at the community level [57]. Studies have shown that N is only limited if N/P < 9, N and P collimate if 9 ≤ N/P < 22.6, and P is only limited if N/P ≥ 22.6 [58].Therefore, WTN/WTP can be used as an important index to judge the risk of eutrophication in water. 

**Figure 9:**
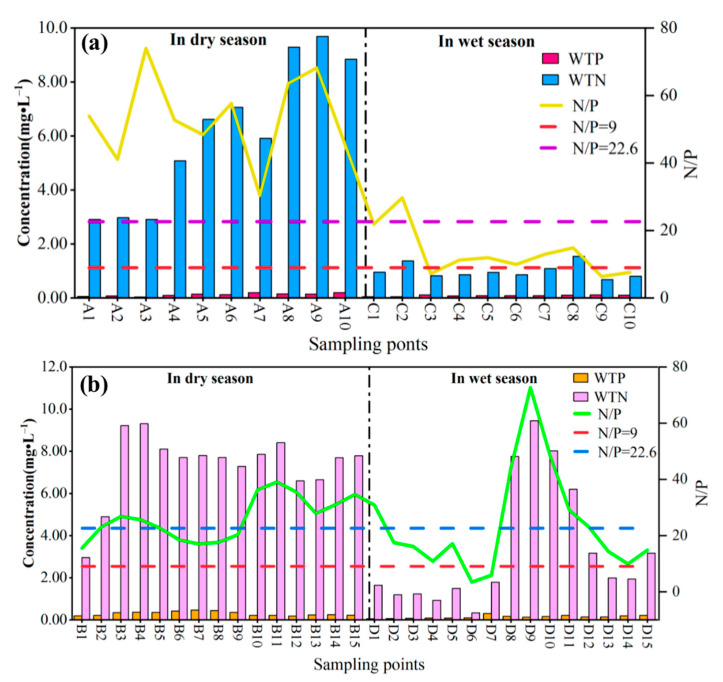
The ratio of N to P (N/P) in the study area ((**a**): Pi river, (**b**): Shiting river).

The results of WTN/WTP in the water of the Pi and Shiting rivers are shown in Figure 9. In our study, the N/P of the Pi river in the dry season was in the range of 30.4–73.9, with an average of 53.5, and the N/P at all sampling points was greater than 22.6. Meanwhile, in the wet season, the N/P was in the range of 6.37–29.7, with an average of 13.4. Among them, the N/P in C3, C9, and C10 was less than 9, and the limiting factor affecting algal growth was N. The N/P at point C2 was greater than 22.6, and the limiting factor affecting algal growth was P. The N/P at the other six sampling points was greater than 9 and less than 22.6, and the limiting factors affecting the growth of algae were N and P. Therefore, P was the main limiting factor affecting the growth of algae in the dry season, while N and P were the main limiting factors in the wet season. In the dry season, the N/P in the Shiting river was in the range of 5.19–83.9, with an average of 30.4, among which the N/P at B3 was 5.19, and those at B6, B7, and B15 ranged from 9 to 22.6. The limiting factors affecting algal growth were N and P. The N/P at the other 11 sampling points were higher than 22.6, and the limiting factor affecting algal growth was P. In the wet season, N/P ranged from 2.61 to 80.4, the average N/P was 22.6, and the values at D6 and D10 were less than 9. The limiting factor affecting algal growth at these points was N. The N/P in D1–D5 and D11–D15 was more than 9 and less than 22.6 and the limiting factors affecting algal growth were N and P. The N/P in the other three sampling points were higher than 22.6, and the limiting factor affecting the growth of algae was P. Therefore, in the Shiting and Pi rivers, the main limiting factor affecting algal growth in the dry season was P, and that in wet season was both N and P. Future management of the Pi and Shiting rivers should focus on the controlling both N and P.

## 4. Source Analysis of Nitrogen and Phosphorus in the Pi and Shiting Rivers

### 4.1. Correlation Analysis

**Figure 10:**
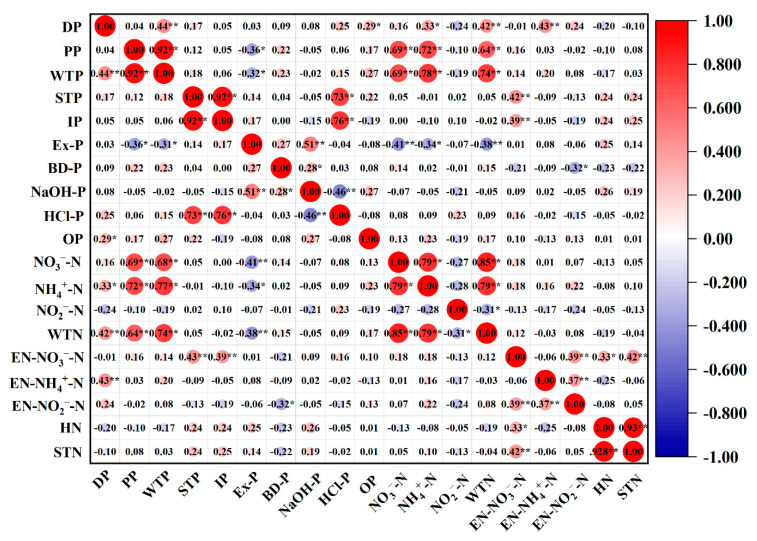
Correlation between different N and P in the study area (**: Correlation is significant at the 0.01 level, *: Correlation is significant at the 0.05 level.).

A Spearman correlation coefficient test was used for correlation analysis (CA); the results are shown in Figure 10. The correlation coefficient showed that PP and DP were significantly correlated with WTP (*p* = 0.01), and PP and DP were the main contributors to WTP. The concentrations of these two fractions of phosphorus directly affected the concentrations of WTP, and PP could be converted into DP, which was then easily absorbed by organisms under certain conditions [39]. However, PP and WTP were significantly correlated with NH_4_^+^-N, NO_3_^−^-N, and WTN (*p* = 0.01), indicating that the concentration of phosphorus in water was affected by NH_4_^+^-N, NO_3_^−^-N, and WTN. These fractions of nitrogen and phosphorus had similar sources. Notably, exogenous pollution was in the form of the decomposition of animal and plant residues and aquaculture wastewater. Both HCl-P and IP, which were relatively stable fractions of phosphorus in surface sediments [51], were significantly correlated with STP (*p* = 0.01), and they were the main contributors to STP. In addition, STP was significantly correlated with EN-NO_3_^−^-N (*p* = 0.01). EN-NO_3_^−^-N was the fraction in the surface sediment which was most prone to releasing NO_3_^−^-N into the water. In conclusion, these fractions may have similar pollution sources. Moreover, STP, HCl-P, and IP may affect the release of NO_3_^−^-N from surface sediments.

Ex-P was significantly correlated with NaOH-P (*p* = 0.01), and BD-P was strongly correlated with NaOH-P (*p* = 0.05). These fractions of phosphorus were greatly affected by point source pollution such as industrial wastewater and domestic sewage [59], and Ex-P, BD-P and NaOH-P can be mutual transformative under certain conditions. All three fractions belonged to the bioavailable phosphorus in surface sediments, which are readily released into water, where they can be directly absorbed and utilized by organisms. The higher the content of these three fractions, the higher the content of active phosphorus in surface sediments, and the greater risk of phosphorus release. In addition, STN was significantly correlated with HN (*p* = 0.01), indicating that HN was the main contributor to STN. The nitrogen in the surface sediments was mainly organic; HN was the main organic fraction of nitrogen [60] and had a great influence on STN. In addition, there was some correlation between EN-NO_3_^−^-N and STN, indicating that agricultural non-point source pollution had some impact on EN-NO_3_^−^-N, STN, and HN levels [61]. EN-NO_3_^−^-N, EN-NO_2_^−^-N, and EN-NH_4_^+^-N, which are more active fractions of nitrogen in surface sediments, had a strong correlation (*p* = 0.05), EN-NH_4_^+^-N tends to release NH_4_^+^-N into water, while EN-NO_3_^−^-N readily releases NO_3_^−^-N. In contrast, EN-NO_2_^−^-N readily releases NO_2_^−^-N into water, and these three fractions can be transformed into each other under the action of microbial nitrification and denitrification [56]. The nitrogen and phosphorus fractions in water were closely related to surface sediments, and the nitrogen and phosphorus pollution in the study area was mainly attributed to industrial wastewater, domestic sewage, and agricultural pollution.

### 4.2. Cluster Analysis

**Figure 11:**
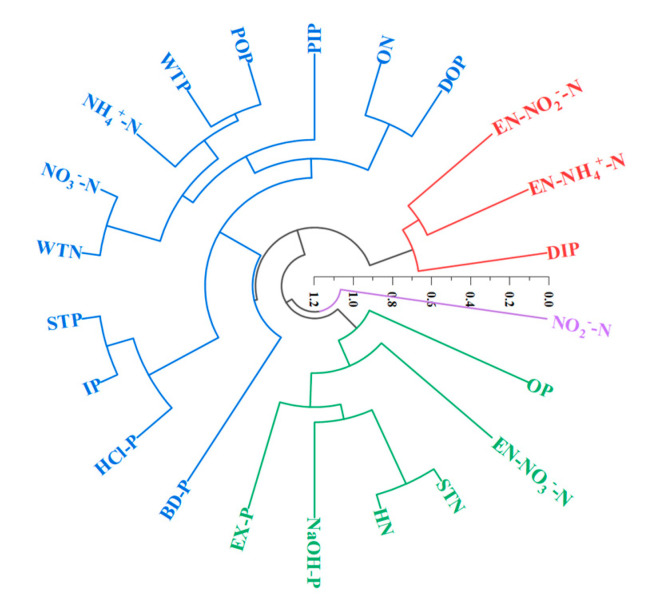
Cluster analysis of N and P in the study area (Different colors represent different classification results).

The strength of the aforementioned correlations reflects the homology of nitrogen and phosphorus. It is generally believed that the higher the correlation, the more likely it is to belong to the same source. However, cluster analysis (CA) classifies the nitrogen and phosphorus with similar sources into one class according to the correlation, and reveals the relationship of different forms of nitrogen and phosphorus via the distances between points in the tree diagram, allowing us to determine the pollution source [62]. Therefore, CA was carried out on the total of nitrogen and phosphorus and their fractions in the water and surface sediments taken from the sampling points in the Pi and Shiting rivers in the dry season and wet season. The results are shown in Figure 11. 

It can be seen from Figure 11 that the CA divided the nitrogen and phosphorus into four categories; among them, DOP, PIP, POP, WTP, NH_4_^+^-N, NO_3_^−^-N, ON, WTN, STP, IP, HCl-P, and BD-P were classified into the first category, EN-NO_2_^−^-N, EN-NH_4_^+^-N and DIP into the second category, OP, Ex-P, NaOH-P, HN, STN and EN-NO_3_^−^-N into the third category, and NO_2_^−^-N into the fourth category. The fractions of nitrogen and phosphorus in each category had similar or identical pollution sources.

In the first category, POP and WTP, ON and DOP, NO_3_^−^-N and WTN, and STP and IP were divided into one group each, which indicated that these fractions of nitrogen and phosphorus had certain relationships with each other. For example, IP was the main contributor of STP, POP, and NO_3_^−^-N, accounting for a large proportion of WTP and WTN respectively, while ON and DOP had similar pollution sources, and both are organic pollutants. At the next level, STP, IP, and HCl-P belonged to the same group. This was because HCl-P and IP were relatively stable fractions of phosphorus in surface sediments which do not readily enter the water, and were the main contributors to STP. Therefore, the first category of nitrogen and phosphorus fraction was mainly affected by agricultural non-point source pollution and industrial wastewater discharge. In the second category, EN-NO_2_^−^-N and EN-NH_4_^+^-N were divided into one group, because these are more active nitrogen fractions in surface sediments which readily release NO_2_^−^-N and NH_4_^+^-N into water. In the next level, these forms were divided into a single group with DIP. DIP was mainly derived from cellular and biological excretion [39]. It can be seen that the three fractions mainly came from aquaculture wastewater, animal excretion, and the decomposition of animal and plant residues. In the third category, HN and STN were first divided into a single group, and then further divided into a group with NaOH-P and Ex-P, respectively. Finally, they were classified into a category with OP. On the one hand, HN accounted for a large proportion to STN, and NaOH-P and Ex-P comprised bioavailable phosphorus in surface sediments. On the other hand, these fractions of nitrogen and phosphorus were greatly affected by industrial wastewater, domestic sewage, and agricultural pollution [63]. The fourth category classified NO_2_^−^-N separately because it is rather rare and does not readily accumulate in water and surface sediments. The reason for this is that the conversion rate of NO_2_^−^-N to NO_3_^−^-N is greater than that of NH_4_^+^-N to NO_2_^−^-N [9], resulting in a low content of NO_2_^−^-N. This form mainly comes from pesticides, fertilizers, and the bait used in the breeding industry. Therefore, based on the results of our CA, it could be inferred that the study area was mainly affected by human activities in the forms of the decomposition of animal and plant residues, aquaculture wastewater, the unreasonable application of pesticides and fertilizers, rural non-point source pollution, and industrial waste discharge.

### 4.3. Principal Component Analysis

**Figure 12:**
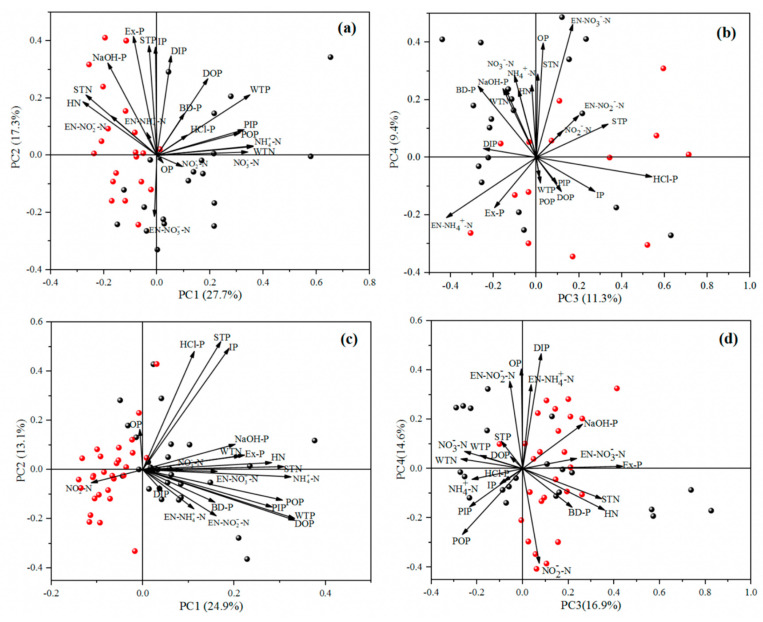
Plane scattered point load map of different N and P based on PCA. (**a**,**b**) show the four principal components extracted in Pi River, (**c**,**d**) show the four principal components extracted in Shiting River.

In this paper, principal component analysis (PCA) was carried out for different nitrogen and phosphorus in water and surface sediments in the Pi and Shiting rivers. The results are shown in Figure 12. The four principal components shown in Figure 12a,b can explain 65.7% of the total data variance in the Pi river. It can be seen from the figure that (1) PC1 (27.7%) is closely related to NH_4_^+^-N, NO_3_^−^-N, and WTN. There are dense urban areas around the Pi river, and the breeding and planting industries there are more developed. Human activities livestock feces increase the concentrations of NH_4_^+^-N, NO_3_^−^-N, and WTN. Thus, it was speculated that the first principal components were domestic sewage and aquaculture wastewater from the surrounding area. (2) PC2 (17.3%) was closely related to STP, IP, Ex-P, and DIP. It was reported in the literature [39] that plankton and bacterial cells can secrete and release DIP, while DP, Ex-P, and STP are related to animal excretion [59]. In addition, the degradation of animal and plant residues was also likely one of the main sources of DP. Therefore, the second principal component was speculated to be related to the decomposition of aquaculture wastewater and animal and plant residues. (3) PC3 (11.3%) had a high correlation with HCl-P and IP, which are inert fractions of phosphorus in surface sediments. Leachate generated from domestic garbage accumulation was one of the main sources of HCl-P and IP [64]. Therefore, it was speculated that the third principal component was related to the leachate produced by the accumulation of domestic garbage. (4) PC4 (9.40%) had a high correlation with OP and EN-NO_3_^−^-N. OP was mainly derived from the decomposition of animal and plant residues and agricultural non-point source pollution, while EN-NO_3_^−^-N was related to leachate produced by the accumulation of domestic waste.

The four principal components in Figure 12c,d explain 69.5% of the total data variance in the Shitting river. According to the figure: (1) PC1 (24.9%) was closely related to NH_4_^+^-N, HN, STN, and WTP. Among them, NH_4_^+^-N is a toxic pollutant which often exists in landfill leachate and other wastes, such as sewage, fertilizer, and other organic pollutants. Organic matter release and fertilizer decomposition in agricultural applications are important sources of HN, NH_4_^+^-N, STN, and WTP [55]. The Shiting river is surrounded by an intensive agricultural cultivation area, and as such, is greatly affected by agricultural non-point source pollution. Therefore, it was speculated that the first principal component was agricultural non-point source pollution. (2) PC2 (13.1%) was closely related to STP, HCl-P and IP. HCl-P and IP are very stable fractions of phosphorus in surface sediments which often exist in inert media such as minerals; they do not readily decompose and enter water. There are a large number of phosphorus chemical enterprises and other industrial areas clustered within the Shiting river basin. Most of the phosphogypsum resulting from the production process accumulates along the river bank, having a great influence on the fraction of phosphorus in the river. Therefore, it was speculated that the second principal component was related to nearby phosphate mines and the discharge of industrial wastewater. (3) PC3 (16.9%) was closely related to EN-NO_3_^−^-N, STN and Ex-P. According to the literature [50], Ex-P is associated with animal excrement in the breeding industry, and the decomposition of livestock excrement and animal and plant residues are one of the main sources of STN and EN-NO_3_^−^-N. Therefore, it was speculated that the third principal component was related to the decomposition of animal and plant residues. (4) PC4 (14.6%) showed a high correlation with OP, DIP, and EN-NH_4_^−^-N. EN-NH_4_^−^-N is ammonia nitrogen which is easily released from surface sediments and is related to agricultural production processes. OP mainly came from agricultural non-point source pollution, and DIP mainly came from plankton excretion [39,64]. In addition, the feces produced by livestock and poultry also increased the concentrations of OP and NH_4_^−^-N. The Shiting river basin is an important agricultural planting base in the Chengdu Plain, and livestock, poultry, and wild animals are widely distributed. Therefore, it was speculated that the fourth principal component was related to agricultural non-point source pollution.

## 5. Conclusions

In this paper, the distribution characteristics of nitrogen and phosphorus in water and surface sediments in the Pi and Shiting rivers in different seasons were studied. The sources of nitrogen and phosphorus pollution in the two rivers were identified by correlation analysis, cluster analysis and principal component analysis. The nitrogen and phosphorus in the water and surface sediments of the Pi and Shiting rivers showed different levels of pollution. In the dry season, the main contributors of WTP were PIP and POP, while in the wet season, they were POP and DIP. NO_3_^−^-N and ON were the main contributors of WTN in the two seasons. P was the main limiting factor of algae growth in the two rivers in the dry season, but N and P were the main limiting factors in the wet season. In addition, in surface sediments, HCl-P and HN were the major contributors to STP and STN, respectively. In the dry and wet seasons, the bioavailable phosphorus (BAP) in the surface sediments of the Pi river posed a higher risk of phosphorus release than that of the Shiting river, and monitoring should be undertaken in that area. However, exchangeable nitrogen, which is more readily released into water, accounted for a large proportion in the surface sediments in the Shiting river. 

The results of our source analysis showed that in the Pi river, the decomposition of animal and plant residues, the leachate generated by aquaculture wastewater, and the accumulation of domestic waste and urban domestic sewage were the main sources of nitrogen and phosphorus pollution in the river. Meanwhile, in the Shiting river, the unreasonable application of pesticides and fertilizers, the degradation of animal and plant residues, wastewater from agricultural drainage channels, wastewater from industrial production, and the weathering of agricultural wastes had a great impact on the nitrogen and phosphorus pollution in the river. Therefore, the relevant local departments should implement control measures according to the different pollution sources in the two rivers in order to reduce the nitrogen and phosphorus pollution load in the river basin.

## Data Availability

Some or all data and models that support the findings of this study are available from the corresponding author upon reasonable request.

## Supplementary Materials

The following supporting information can be downloaded at: https://www.mdpi.com/article/10.3390/ijerph191912433/s1. Table S1. The standard of surface water environmental quality in China. Table S2. Specification for eutrophication investigation in China. Table S3. Evaluation standard of nitrogen and phosphorus pollution in sediments. Table S4. The concentrations of total nitrogen and phosphorus and their fractions concentration in water. Table S5. The content of total nitrogen and phosphorus and their fractions in surface sediments.
