# Supplementary material for: Distribution Characteristics and Source Analysis of Nitrogen and Phosphorus in Different Rivers in Two Water Period: A Case Study of Pi River and Shiting River in the Upper Reaches of Tuo River in China
Source: Int J Environ Res Public Health. 2022 Sep 29;19(19):12433. doi: 10.3390/ijerph191912433 (PMC9566003; doi:10.3390/ijerph191912433)
Supplement: Supplementary file 1 [file ijerph-19-12433-s001.zip › ijerph-1921420 - supplementary.pdf]

International Journal of
Environmental Research and Public Health

MDPI

The Standards and Specification of Surface Water Environmental Quality, Eutrophication and Pollution Degree of Nitrogen and Phosphorus in Sediments

Table S1. The standard of surface water environmental quality in China.

Standard Value	Class I	Class II	Class III	Class IV	Class V
TP(mg·L-1)	≤0.010	0.010~0.025	0.025~0.050	0.050~0.100	0.100~0.200
TN(mg·L-1)	≤0.200	0.200~0.500	0.500~1.00	1.00~1.50	1.50~2.00

Table S2. Specification for eutrophication investigation in China.

Standard Value	Hypotrophic Type	Medium Trophic Type	Eutrophic Type	Heavy Eutrophic Type
TP(mg·L-1)	<0.020	0.020~0.050	0.050~0.090	>0.090
TN(mg·L-1)	<0.250	0.250~0.700	0.700~1.30	>1.30

Table S3. Evaluation standard of nitrogen and phosphorus pollution in sediments.

Evaluation Criterion	Content of STP (mg·kg-1)	Content of STP (mg·kg-1)	Pollution Degree
The standards formulated by the US EPA	<420	<1000	Mildly polluted
	420-~650	1000~2000	Moderately polluted
	>650	>2000	Severely polluted
The standards formulated by Environment Canada (DOEE)	<600	<550	Safety ecotoxic effect
	600~2000	550~4800	The lowest ecotoxic effect
	>2000	<4800	Serious ecotoxic effect

Experimental Data in This Study

Table S4. The concentrations of total nitrogen and phosphorus and their fractions concentration in water.

Water Period	Sampling Point	Total Phosphorus and Phosphorus Fractions in Water (mg·L-1)							Total Nitrogen and Nitrogen Fractions in Water (mg·L-1)				
		WTP	DP	PP	DIP	DOP	PIP	POP	WTN	NO_3^--N	NH_4^+-N	NO_2^--N	ON
In the dry season	A1	0.0540	0.021	0.033	0.010	0.011	0.025	0.0081	2.91	1.08	0.24	0.0085	1.58
	A2	0.0723	0.042	0.030	0.037	0.0050	0.013	0.017	2.97	1.06	0.415	0.016	1.48
	A3	0.0394	0.028	0.012	0.026	0.0018	0.0077	0.0038	2.91	1.01	0.436	0.017	1.45
	A4	0.0964	0.025	0.071	0.019	0.0063	0.018	0.053	5.08	1.72	0.824	0.025	2.52
	A5	0.137	0.028	0.11	0.018	0.011	0.074	0.035	6.61	2.34	0.759	0.030	3.49
	A6	0.123	0.032	0.091	0.028	0.0038	0.023	0.068	7.06	2.47	0.878	0.030	3.68
	A7	0.195	0.039	0.16	0.022	0.017	0.065	0.090	5.91	1.70	0.933	0.028	3.25
	A8	0.146	0.087	0.059	0.069	0.018	0.013	0.047	9.29	3.20	0.775	0.045	5.27
	A9	0.142	0.080	0.062	0.067	0.013	0.028	0.034	9.68	3.57	0.960	0.040	5.11
	A10	0.195	0.11	0.088	0.086	0.021	0.055	0.033	8.84	3.14	0.705	0.050	4.95
	B1	0.0509	0.029	0.022	0.027	0.0016	0.013	0.0093	2.28	0.722	0.162	0.025	1.37
	B2	0.151	0.11	0.045	0.10	0.0023	0.011	0.034	3.42	1.23	0.488	0.028	1.68
	B3	0.776	0.30	0.47	0.17	0.13	0.25	0.22	4.03	1.39	0.756	0.010	1.87
	B4	0.255	0.057	0.20	0.040	0.017	0.11	0.087	6.49	2.03	1.08	0.013	3.37
	B5	0.358	0.073	0.28	0.034	0.039	0.097	0.19	8.63	2.20	2.02	0.065	4.34
	B6	0.417	0.049	0.37	0.018	0.031	0.11	0.26	7.70	2.03	1.65	0.012	4.00
	B7	0.482	0.047	0.43	0.026	0.021	0.27	0.16	8.63	2.51	1.72	0.011	4.38
	B8	0.217	0.060	0.16	0.046	0.014	0.084	0.073	7.85	1.28	1.49	0.011	5.07
	B9	0.179	0.085	0.093	0.065	0.020	0.048	0.045	6.38	2.45	0.897	0.007	3.02
	B10	0.280	0.15	0.13	0.12	0.024	0.069	0.065	6.75	2.51	0.946	0.022	3.27
	B11	0.215	0.064	0.15	0.042	0.023	0.071	0.079	7.03	2.50	1.04	0.012	3.48
	B12	0.196	0.076	0.12	0.055	0.021	0.044	0.076	7.39	2.46	1.09	0.011	3.83
	B13	0.111	0.047	0.063	0.033	0.014	0.030	0.033	9.28	3.25	0.949	0.035	5.05
	B14	0.334	0.22	0.12	0.18	0.043	0.029	0.086	8.89	1.58	1.41	0.056	5.84
	B15	0.364	0.23	0.14	0.18	0.052	0.053	0.083	7.56	2.42	1.85	0.032	3.26
In the wet season	C1	0.0435	0.018	0.025	0.013	0.0058	0.0060	0.019	0.953	0.0223	0.241	0.016	0.673
	C2	0.0462	0.024	0.023	0.023	0.0010	0.016	0.0067	1.37	0.681	0.211	0.047	0.431

C3	0.114	0.10	0.012	0.095	0.0068	0.0077	0.0043	0.825	0.344	0.341	0.011	0.127
C4	0.0770	0.033	0.044	0.018	0.014	0.0091	0.035	0.864	0.293	0.187	0.012	0.372
C5	0.0796	0.024	0.055	0.017	0.0068	0.017	0.039	0.950	0.384	0.303	0.0049	0.257
C6	0.0864	0.021	0.066	0.018	0.0021	0.029	0.037	0.859	0.035	0.260	0.012	0.551
C7	0.0844	0.023	0.062	0.020	0.0026	0.0091	0.053	1.09	0.026	0.266	0.025	0.774
C8	0.104	0.050	0.053	0.046	0.004	0.015	0.038	1.54	1.15	0.374	0.0040	0.0121
C9	0.107	0.078	0.029	0.034	0.044	0.010	0.019	0.681	0.00134	0.290	0.012	0.378
C10	0.106	0.081	0.025	0.049	0.032	0.0077	0.017	0.804	0.222	0.293	0.0049	0.284
D1	0.0687	0.022	0.047	0.014	0.0078	0.0084	0.039	1.42	0.808	0.450	0.013	0.153
D2	0.0718	0.025	0.047	0.018	0.0063	0.018	0.029	1.20	0.731	0.279	0.033	0.154
D3	0.0854	0.035	0.051	0.028	0.0068	0.016	0.035	1.38	0.437	0.279	0.016	0.646
D4	0.100	0.028	0.072	0.019	0.0094	0.036	0.036	1.72	0.442	0.225	0.18	0.878
D5	0.0870	0.026	0.061	0.023	0.0026	0.038	0.023	1.48	0.588	0.228	0.027	0.639
D6	0.683	0.653	0.030	0.59	0.061	0.025	0.0055	1.78	0.505	0.485	0.0055	0.786
D7	0.130	0.024	0.11	0.017	0.0068	0.039	0.067	10.4	3.99	0.350	0.22	5.88
D8	0.168	0.11	0.054	0.11	0.0005	0.021	0.033	6.31	0.0665	0.298	0.010	5.94
D9	0.180	0.15	0.030	0.14	0.012	0.028	0.0018	7.39	1.79	0.301	0.019	5.28
D10	0.158	0.073	0.085	0.062	0.012	0.0084	0.076	1.40	0.507	0.385	0.026	0.481
D11	0.214	0.16	0.050	0.16	0.0042	0.041	0.0088	2.91	0.954	0.382	0.042	1.53
D12	0.190	0.11	0.076	0.098	0.016	0.040	0.037	1.99	0.663	0.322	0.0029	1.00
D13	0.188	0.17	0.023	0.15	0.015	0.011	0.012	3.33	0.325	0.515	0.0055	2.49
D14	0.153	0.048	0.11	0.037	0.011	0.039	0.066	2.65	0.888	0.369	0.16	1.23
D15	0.174	0.060	0.11	0.056	0.0047	0.051	0.063	3.68	0.785	0.390	0.021	2.49

Table S5. The content of total nitrogen and phosphorus and their fractions in surface sediments.

Water Period	Sampling Point	Total Phosphorus and Phosphorus Fractions in Surface Sediments (mg·kg^{-1})						Total Nitrogen and Nitrogen Fractions in Surface Sediments (mg·kg^{-1})				
		**STP**	**Ex-P**	**BD-P**	**NaOH-P**	**HCl-P**	**OP**	**STN**	**EN-NO_3^--N**	**EN-NH_4^+-N**	**EN-NO_2^--N**	**HN**
In the dry season	A1	898.6	0.842	21.99	171.1	682.1	421.2	596.5	21.29	45.28	0.35	403.8
	A2	921.3	2.12	87.70	94.75	659.8	377.2	793.0	30.56	27.79	0.42	654.9
	A3	1157	1.00	66.32	192.2	784.2	386.6	1279	47.86	38.09	0.71	1158
	A4	1163	6.89	63.76	153.4	766.2	441.9	833.7	22.25	63.18	0.39	734.9
	A5	1350	6.70	45.31	146.0	812.7	754.7	862.7	13.50	26.57	0.43	810.2
	A6	821.5	1.13	39.89	109.7	551.7	187.3	756.7	15.63	23.31	0.29	594.5
	A7	1195	5.34	49.51	155.2	757.3	288.2	909.6	32.97	33.21	0.89	780.9
	A8	1294	9.02	54.49	298.5	596.6	293.7	1536	26.43	42.98	1.1	1458
	A9	1310	10.5	68.67	275.0	574.5	453.0	769.0	18.10	45.55	0.73	672.9
	A10	1280	10.0	77.52	170.5	833.6	226.3	767.3	8.616	39.99	0.29	662.1
	B1	2219	4.69	38.21	25.75	1411	373.9	1342	14.35	29.80	0.40	1096
	B2	1439	31.4	43.16	51.88	1028	57.78	2131	71.43	87.36	0.53	940.0
	B3	1517	15.7	116.5	228.4	812.3	295.4	1386	20.78	86.68	0.93	980.7
	B4	1378	15.6	53.10	125.2	777.2	229.4	1316	38.68	62.65	1.4	800.1
	B5	1422	2.05	84.93	61.30	1105	159.8	2282	28.35	124.8	1.6	3005
	B6	1580	6.76	40.12	115.0	1144	52.33	737.2	4.799	39.17	0.41	452.4
	B7	1894	6.39	39.77	90.26	1684	109.4	650.7	7.277	40.93	1.5	370.3
	B8	1641	5.18	111.4	146.5	1068	145.1	642.9	19.64	29.93	0.68	434.1
	B9	1691	10.3	95.32	195.7	1075	537.8	1018	14.91	24.37	2.2	592.2
	B10	2861	3.87	22.71	89.72	1708	478.6	468.2	7.790	37.13	0.56	263.0
	B11	1619	4.74	33.94	124.6	1095	116.8	1025	23.44	45.55	0.81	768.0
	B12	1826	4.01	32.87	97.34	1360	276.3	671.9	7.500	31.97	0.70	441.4
	B13	1623	3.72	82.76	99.29	1100	264.7	583.7	11.58	152.3	1.0	235.1
	B14	1554	3.54	47.10	125.3	1086	197.4	761.7	15.76	154.0	1.6	319.5
	B15	1595	7.12	36.44	108.9	1298	169.6	1083	16.43	147.6	2.7	744.8
In the wet season	C1	1085	8.11	12.67	57.57	844.2	222.2	716.5	29.29	7.81	0.42	661.7
	C2	1024	3.06	15.19	100.5	708.5	80.48	1039	8.527	36.04	0.51	975.5
	C3	1400	22.1	55.49	196.0	844.9	157.9	1541	2.254	26.68	0.53	1494

C4	975.3	5.73	23.76	168.2	613.6	77.57	1080	24.46	68.76	1.2	961.4
C5	1312	9.06	32.47	282.9	604.5	343.9	1414	12.50	30.89	0.58	1285
C6	1281	10.2	26.38	152.3	867.2	154.3	915.7	10.63	37.81	0.37	834.6
C7	1428	17.6	49.43	400.2	563.6	335.7	1346	15.45	52.61	0.57	1197
C8	887.3	17.1	41.38	175.2	459.2	30.88	919.6	6.049	31.70	0.38	869.8
C9	1067	15.7	52.49	229.8	439.4	279.7	1105	5.223	44.87	0.78	1025
C10	1818	13.1	54.83	313.6	986.4	367.3	1087	16.47	20.57	1.2	829.5
D1	1312	1.23	91.43	21.51	1163	71.67	422.4	9.777	28.44	0.52	328.7
D2	950.4	5.66	65.08	201.5	579.0	90.14	542.4	3.125	41.75	0.62	496.3
D3	1502	2.23	65.12	26.49	1380	179.5	599.9	11.94	91.30	0.29	491.8
D4	1569	5.58	65.10	50.55	1411	90.61	500.6	8.304	47.99	0.19	426.3
D5	1226	4.06	45.39	79.08	977.2	147.4	419.1	7.054	107.7	1.6	215.3
D6	1107	11.6	45.40	94.02	752.5	302.2	447.5	3.594	109.7	2.1	164.9
D7	1158	7.25	58.23	183.3	742.8	110.8	675.8	4.442	34.14	0.55	525.7
D8	2547	17.4	49.57	351.7	1733	456.9	865.0	12.37	55.73	0.42	519.7
D9	1580	12.5	44.14	223.1	896.3	368.0	742.2	13.66	55.86	0.85	597.3
D10	1543	14.8	55.31	164.8	999.3	133.8	609.4	5.938	34.41	0.45	431.5
D11	1979	21.5	52.36	170.9	1697	43.84	493.9	8.594	102.6	0.66	401.9
D12	1748	14.5	39.79	201.2	1269	391.2	689.7	17.12	107.8	0.82	466.7
D13	1662	8.98	48.35	134.3	1310	69.63	440.3	16.74	85.19	0.36	323.3
D14	1119	10.7	32.79	116.6	882.1	55.06	608.3	13.66	31.97	0.30	508.6
D15	1478	1.47	34.75	96.09	1131	399.0	467.1	6.027	116.1	0.34	339.8
